# Adapting Family Planning Service Delivery in Title X and School-Based Settings during COVID-19: Provider and Staff Experiences

**DOI:** 10.3390/ijerph20043592

**Published:** 2023-02-17

**Authors:** Andrea Vazzano, Sydney Briggs, Lisa Kim, Jenita Parekh, Jennifer Manlove

**Affiliations:** Child Trends, 7315 Wisconsin Avenue, Suite 1200W, Bethesda, MD 20814, USA

**Keywords:** COVID-19, family planning, reproductive health, health services, telehealth, school-based health

## Abstract

The COVID-19 pandemic introduced urgent and unique challenges to family planning providers and staff in ensuring continued access to high-quality services, particularly for groups who experience greater barriers to accessing services, such as women with systemically marginalized identities and adolescents and young adults (AYA). While research has documented key adaptations made to service delivery during the early phase of the pandemic, limited studies have used qualitative methods. This paper draws on qualitative interview data from family planning providers and staff in Title-X-funded clinics and school-based clinics—two settings that serve populations that experience greater barriers to accessing care—to (a) describe the adaptations made to service delivery during the first year of the pandemic and (b) explore provider and staff experiences and impressions implementing these adaptations. In-depth interviews were conducted with 75 providers and staff between February 2020 and February 2021. Verbatim transcripts were analyzed via inductive content analysis followed by thematic analysis. Four key themes were identified: (1) Title-X- and school-based staff made multiple, concurrent adaptations to continue family planning services; (2) providers embraced flexibility for patient-centered care; (3) school-based staff faced unique challenges to reaching and serving youth; and (4) COVID-19 created key opportunities for innovation. The findings suggest several lasting changes to family planning service delivery and provider mindsets at clinics serving populations hardest hit by the pandemic. Future studies should evaluate promising practices in family planning service delivery—including telehealth and streamlined administrative procedures—and explore how these are experienced by diverse patient populations, particularly AYA and those in areas where privacy or internet access are limited.

## 1. Introduction

The COVID-19 pandemic introduced urgent and unique challenges to family planning providers and staff in ensuring continued access to high-quality services. In the first year of the pandemic, providers across the globe navigated service delivery amid concerns over staff and patient safety, rapid introduction of new technology systems, and staffing shortages during a time when much was unknown about the SARS-CoV-2 virus [1,2,3]. Fears were raised early on about implications for access to family planning care [4,5,6], and it has since been well documented that access to sexual and reproductive health (SRH) services declined significantly worldwide [7,8]. Many family planning clients have faced barriers to accessing care, including longer wait times for appointments, fear of entering medical settings, prescription shortages, clinic closures, and increased economic hardship [9,10,11,12].

The effects of COVID-19 on access to family planning services have not been experienced uniformly across all groups and populations and have served to exacerbate existing inequities in family planning care. In the United States, certain population groups—including women with systematically marginalized identities, women living in poverty, and adolescents and young adults (AYA; referring to young people from onset of puberty through age 25)—have long been shown to experience greater barriers to accessing medical care, including SRH services [13,14,15]. These barriers have been heightened in the current context due to the disproportionate, multi-layered impacts of the pandemic [4,9,16,17]. For example, women who face systematic marginalization on the basis of race, ethnicity, and class, including the Black and Hispanic communities, have been disproportionately likely to experience loss of employment and income, in addition to increases in childcare responsibilities, all of which can further impede access to care [18]. Likewise, many AYA in the same communities have experienced their parents’ changing economic circumstances in combination with school closures, leading to a loss of privacy necessary for confidential sexual health care [4,19,20].

Two key mechanisms for providing SRH care to populations that experience greater barriers to accessing services are Title-X-funded clinics and school-based health services. Title X is the only federal grant program dedicated solely to providing low-income or uninsured individuals with comprehensive family planning and related preventive health services, making it an important source of care for individuals from systematically marginalized populations [21,22]. One-quarter of all women, and one-half of women with incomes below the federal poverty level, access contraceptive services at publicly funded family planning centers, with Title-X-funded sites providing the majority of publicly funded care [23]. Title X clients are also more likely to be Black or Hispanic and live under the federal poverty line than the general US population [24,25,26]. Meanwhile, school-based clinics provide on-site services, including SRH care, to students in over 10,000 K–12 schools across the country. These clinics are an important source of SRH care in communities that may otherwise experience limited access to services, especially for young people from low-income and rural populations, and populations of color [26]. School-based clinics can play a critical role in facilitating young people’s transition from pediatric care to the adult health care system, when rates of unintended pregnancy are the highest [27]. The presence of health care services on school campuses also reduces the unique barriers that AYA face to accessing family planning care, such as lack of privacy or access to transportation [28,29].

The emergence of COVID-19 disrupted the ability of clinics—including Title-X-funded and school-based clinics—to offer in-person family planning services [19,30,31]. In 2020, over 77 percent of certified school-based health centers (SBHCs) shut down due to school closures [19], and Title-X-funded clinics saw almost 900,000 fewer clients than before the pandemic [32]. A growing body of survey research has documented the ways providers and staff responded to early disruptions from the pandemic, identifying immediate and ongoing adaptations made to clinical practices and protocols [9,33,34,35,36,37]. In general, there is consensus that providers made rapid transitions to telehealth services, began offering select family planning services in a “drive by” or “curbside” fashion, and implemented updated clinical guidance on contraceptive refills and spacing [9,24,33]. Surveys have provided quantitative data on the prevalence of these adaptations in settings that serve both adults and AYA [35,36,38,39]. For example, one study saw an increase in telehealth use from 11 percent to 79 percent among reproductive health providers [38], and another found that 82 percent of clinics providing abortion or contraceptive care had added or expanded telehealth services for contraceptive counseling [36]. Other service adaptations appear to be less common, with 10 percent to 23 percent of providers surveyed offering self-administered medroxyprogesterone acetate [36,38], 15 percent to 22 percent offering curbside pick-up for contraceptives [36,38], and 15 percent to 35 percent mailing out contraceptives or using mail-order pharmacies [36,38]. Researchers have also surveyed providers on perceptions of the effectiveness, acceptability, and challenges of telemedicine, the most widespread and common change to family planning service delivery during the pandemic [40,41,42]. In one study, Stifani et al. found that 80 percent of family planning providers strongly agreed that telehealth was effective for contraceptive counseling, and a majority supported continuing to provide it post-pandemic, with a significant preference for video visits over phone visits [40]. This and other studies have also shed light on key limitations of telehealth, including not being able to conduct physical exams or diagnostic testing, patient difficulties using telehealth, insufficient cellular service or poor Wi-Fi connections, and disparities in access to technology [40,41]. Additional challenges reported by providers at youth-serving clinics include lack of client awareness of telehealth services and concerns around confidentiality [42].

Although a considerable amount of scholarship has explored family planning providers’ responses and adaptations to continue providing care, few published studies have included qualitative data and methods. Developing a qualitative understanding of not only how family planning providers and staff modified care but also their impressions and experiences of these changes can offer critical information to researchers and practitioners looking to ensure high-quality services, particularly for populations hardest hit by the pandemic. To date, limited qualitative studies have examined family planning service adaptations during COVID-19. These studies include a textual analysis of Title-X-funded clinic progress reports documenting reported service delivery adaptations [37], a content analysis of open-ended survey responses from providers implementing telehealth services for contraceptive care [42], and a case study of rapid implementation of telehealth across multiple specialties at a clinic serving AYA [2]. While these efforts have helped to define the changes made to family planning practice in various settings, they do not provide an in-depth exploration of provider experiences with a range of adaptations. Only one recent report—a descriptive study of private, hospital-affiliated, and Planned-Parenthood-affiliated clinics providing abortion or contraception services—has explored this topic qualitatively through in-depth interviews with providers [43]. Ly et al. found that providers faced multiple challenges to modifying abortion and contraception services during COVID-19, including staffing and limited resources, but saw rewards to doing so, such as increased camaraderie and creativity among staff. Although this study provides important context, there remains a need to better understand the experiences of family planning providers more broadly, particularly in settings that serve populations facing barriers to accessing care.

This paper seeks to build on and expand existing research by exploring family planning provider experiences adapting services during COVID-19 in Title-X-funded and school-based clinics—two settings serving women and AYA facing limited access to SRH services. Through content and thematic analysis of in-depth interviews, we aim to (a) describe the adaptations made to family planning service delivery in these settings during the first year of the COVID-19 pandemic and (b) explore provider and staff experiences and impressions implementing these adaptations.

## 2. Materials and Methods

### 2.1. Study Background and Design

Data for this analysis come from two sets of qualitative interviews conducted with providers and staff at Title-X-funded clinics and school-based health centers from February 2020 through February 2021. These interviews were designed and implemented under two concurrent projects that shared a principal investigator and key research team members. One project sought to explore trends in publicly funded family planning services through an analysis of publicly available data and interviews with Title X providers and staff. The second project aimed to identify and explore unique or innovative approaches to family planning service delivery in school settings through interviews with family planning providers and staff.

While these projects were distinct in their populations and settings of interest—one focused on providers serving clients of any age in Title X clinics and the other on providers serving AYA in educational settings—both engaged family planning providers and staff for interviews as the pandemic gave way to state-ordered shutdowns. Accordingly, in April 2020, both teams added questions to their interview protocols to capture the ongoing changes made to family planning services due to COVID-19. Questions were similar across the two studies and explored how clinics modified their services in response to the pandemic, how well these strategies met the needs of their clients, and what challenges they faced in making these changes. Given the overlap in these questions, the authors combined these data to enable a more comprehensive view of the family planning landscape during COVID-19. This paper reports on this subset of COVID-19-related data; broader findings from each specific project have been disseminated elsewhere [44,45,46].

### 2.2. Study Recruitment and Enrollment

Teams followed two separate recruitment protocols under the two projects. For interviews with Title X providers and staff, the research team began recruitment by accessing publicly available lists of Title X sites. Team members stratified clinics by key variables, including geographic region, urban–rural classification, and racial/ethnic composition of client population, and then randomly selected 150 clinics for recruitment via email. Based on initial responses and interest, the team then reached out to an additional 33 clinics that were located in geographic regions where recruitment lagged. Screening interviews were administered to staff at each clinic that was responsive to recruitment to determine eligibility and willingness to participate. Eligibility criteria included having received Title X funding within the past two years and serving over 50 family planning clients per year. The team ultimately enrolled and conducted in-depth interviews with 46 providers and staff from current and former Title X clinics. Of those, 38 were asked questions relating to COVID-19 (i.e., conducted after April 2020) and were, therefore, included in this analysis (see Table 1 for study sample characteristics). These 38 interviewees represented 33 unique sites.

For the second project, the research team used a snowball sampling approach to identify and enroll school-based providers and staff. This approach was selected as the project focused on identifying and exploring novel or “innovative” approaches to family planning service delivery in school settings, which required referrals from practitioners. Eligibility criteria for interviews included operating within or in partnership with a school setting, serving one or more population groups that experience greater barriers to accessing SRH care (i.e., people of color, including members of American Indian Tribes; people with limited English proficiency; people who have immigrated to the United States; people experiencing or at risk of experiencing homelessness; people with low incomes; rural communities; and communities without family planning clinics), and implementing an innovative approach to providing family planning care to AYA in school settings. For the purposes of the project, “school settings” were defined as clinics located within K–12 schools, provider partnerships with K–12 schools, and clinics located at community colleges, which are more likely to serve students from systematically marginalized identities [47,48]. Additionally, for the purposes of the project, “innovative approach” was defined as an intentional and focused approach that reaches one or more population groups experiencing greater barriers to accessing services. To identify potential interviewees, the team first reached out to key contacts, such as regional coordinators of certified SBHCs, and asked them to provide names and contact information for providers and/or clinics that might meet eligibility criteria. The team then conducted a round of screening interviews and focus groups to identify sites best suited for in-depth interviews. Ultimately, the team enrolled and conducted in-depth interviews with 57 school-based providers and staff, representing 48 unique sites. Of the 57 participants, 37 (representing 32 unique sites) were asked questions relating to COVID-19 and were included in this analysis (Table 1).

Table 1 shows key characteristics of participants and associated clinics for the 75 interviews with Title X and school-based staff included in this analysis. In addition, of the Title X clinics interviewed, 45% served at least 20% Hispanic clients and 39% served at least 20% Black clients. The team did not record information on populations served by school-based staff.

**Table 1:** Interviewee and clinic characteristics.

	Title X Staff	School-Based Staff	Total
	*n* = 38	*n* = 37	*n* = 75
**Geographic Location of Clinic**			
Northeast	9	7	16
Midwest	9	8	18
South	11	10	21
West	9	12	21
**Service Location of Clinic**			
Urban	26	32	58
Rural	12	5	18
**Interviewee Role**			
Medical Provider	12	15	27
Administrator	12	13	25
Both Medical Provider and Administrator	12	4	16
Other Service Provider *	2	5	7

* Includes health educator, social worker, and licensed professional counselor.

### 2.3. Data Collection

For both projects, in-depth, semi-structured interviews were conducted via encrypted Microsoft Teams teleconferencing software (version 1.5) and lasted approximately 60 to 90 min. Two trained research team members were present at each interview. Prior to being interviewed, all participants were informed of the study purpose, the voluntary nature of their participation, their rights to withdraw participation at any time, and the confidentiality of their responses. Interviews were audio-recorded with permission from participants. Audio recordings were transcribed verbatim by an outside vendor and supplemented with notes taken by research team members. Participants received a USD 25 gift card for their participation.

### 2.4. Data Analysis

Verbatim transcripts were uploaded into Dedoose [49], a qualitative analysis software, for formal coding. For the purposes of this analysis, three research team members, each of whom participated in data collection for one or both studies, coded only portions of the interviews related to the COVID-19 pandemic. The team approached data analysis in two stages. First, analysts utilized an inductive content analysis approach to identify common adaptations discussed in the interview excerpts [50]. Inductive content analysis is a helpful means of reducing and grouping data according to categories identified in the data themselves [51]. Analysts first read through all the COVID-related excerpts to familiarize themselves with the data and worked together to generate an initial codebook based on the adaptations discussed by participants. Each analyst then independently coded a set of excerpts with the aim of sorting data into distinct categories of adaptations. The team met often to review coding and come to consensus when discrepancies arose [52]. Following this, the team revisited the coded data, analyzing excerpts thematically to generate themes on provider experiences and impressions. Thematic analysis is a flexible, iterative approach to qualitative research that is well suited to understanding a set of experiences and impressions across a dataset [53,54]. Analysts followed documented guidelines in thematic analysis, independently conducting open coding and searching for initial themes before coming together to compare codes, review themes, and achieve consensus on findings [53,55]. The team conducted multiple rounds of review, refinement, and comparison against the data before finalizing the most salient themes.

## 3. Results

Excerpts from interviews with staff at Title-X-funded clinics and school-based health centers revealed four key themes around family planning service delivery and experiences in the first year of COVID-19. These are described in detail below and can be summarized as follows: (1) Title X and school-based staff made multiple, concurrent adaptations to continue family planning services; (2) providers embraced flexibility for patient-centered care; (3) school-based staff responded to unique challenges to reach and serve youth; and (4) COVID-19 created key opportunities for innovation.

### 3.1. Title X and School-Based Staff Made Multiple, Concurrent Adaptations to Continue Family Planning Services

Providers and staff at both Title X and school-based clinics described making multiple adaptations to family planning service delivery to meet ongoing patient needs in the early months of the COVID-19 pandemic. The specific service adaptations described by providers and staff fell into five categories. These include: (1) utilizing telemedicine, including conducting contraceptive counseling and follow-up visits by phone or video call; (2) prioritizing urgent services for in-person care, such as colposcopies and LARC insertions, while delaying or remotely delivering non-urgent services; (3) providing select services “curbside,” including delivering prescriptions or contraceptive injections outside or in a “drive-through” fashion; (4) adopting flexible approaches to birth control refills and spacing, such as prolonging the time required between contraceptive injections and in-office visits for oral contraceptive refills; and (5) using phone or digital technology to streamline administration, including digitizing forms and performing intakes over the phone.

While each strategy was distinct, it was rare to hear from clinics making only one or two changes to practice. Instead, providers and staff tended to report making multiple, concurrent adaptations to reduce the number of people physically entering the clinic and ensure the safety of those onsite. Many providers and staff described several overlapping changes, illustrating how these adaptations worked together and how no one service adaptation was adequate to keep services up and running. For example, one Title X provider explained how they used telehealth, curbside services, flexible birth control refills, and streamlined administration to accommodate patients:

*For every appointment, whether it’s a telehealth appointment or an in-person appointment, our healthcare techs call a patient ahead of time and complete all their history forms with them by phone trying to reduce the amount of time that they spend here in the building. For Depo, we’re now having them come in to get their Depo, but for a little while now, we were going up to their car. For birth control pills, I will say until about June, we would just do a quick phone call and six-month refill to make sure everything’s okay… We’re now doing telehealth visits for pill refills, and we try [to give] just as many as we can allowable by the expiration date.*

In another instance, a Title X clinic administrator discussed their current intake process for LARC appointments, which uses both curbside services and streamlined administrative procedures to prepare clients for procedures:

*So now the client pulls into our parking lot. We have an iPad that we use as a kiosk for check-in. A staff member goes out to their vehicle, takes their temperatures, gives them an iPad. They do the registration and fill out a health questionnaire … That questionnaire gets imported into their medical record. I review that, call them, go over any information that needs clarification, put in their medications, drug allergies, do sort of that telephone intake. And then typically, I’ll just transfer that phone call to the provider who speaks with them from a different part of our clinic and goes over all of the risks and benefits, contraceptive counseling, and once they’ve done that…goes over the consent for the procedure.*

The individual service delivery adaptations are described in greater detail in Table 2, which summarizes these adaptations and describes provider and staff impressions, along with illustrative quotes, for each.

### 3.2. Providers Embraced Flexibility for Patient-Centered Care

Providers from both Title X and school-based clinics emphasized that the pandemic required immediate and ongoing flexibility in how they responded to the changing pandemic environment and in the way they worked with patients. Throughout the early months of the COVID-19 pandemic, providers described needing to make continuous adjustments to their services to ensure that patients received critical services and to maintain quality of care. As one Title X provider stated, *“The first word that comes to mind is ‘constantly.’ Every week is a new adjustment or micro-adjustment to what we put in place the week before.”*

At the start of the pandemic, clinic efforts were largely focused on responding to national guidance on minimizing exposure to COVID-19, which included triaging in-person appointments, developing new clinic protocols, and setting up telehealth services. As described above, many clinics reduced or eliminated non-urgent care and had to prioritize certain services, such as LARC insertions, due to staffing shortages and physical distancing requirements. Clinics also rapidly created new safety protocols, with one provider saying, *“everything was just happening so quickly—it’s been the testing and protocols and cleaning staff and cleaning supplies.”* In addition, many clinics that did not have telehealth systems in place were forced to quickly develop and implement a means for providing remote services. One provider said,

*We knew that telehealth was way off on the horizon for us until it was staring us in the face, so we just need[ed] to put a ton of energy in a short amount of time in getting our telehealth system integrated into our EHR [electronic health record].*

As the pandemic persisted, providers continued to adjust their services to meet the changing landscape of COVID-19. This shift was most evident in the way providers described how telemedicine for family planning services moved from emergency telehealth services only to a more sustainable mix of telehealth and in-person services. One school-based provider said, *“As we learned more about the virus and got infection prevention protocols in place, we started to kind of readjust [the] mix of on-site, face-to-face versus telemedicine.”* In addition, many staff at clinics that did not have an integrated telehealth system in place described first providing services via phone, which was easier to implement, and then ultimately shifting to video telehealth.

This focus on flexibility also extended to the patient level as providers prioritized care that was patient-driven and centered. Providers and staff spoke at length about making on-the-spot adjustments to attend to individual needs. For example, one Title X provider shared an anecdote of a patient who wanted to cancel her annual visit because she had a high-risk family member at home and feared COVID-19 exposure. The patient was scheduled to be seen in person to receive a refill for her birth control pills, so the provider prescribed an additional three months of birth control pills without requiring the in-person appointment and asked her to return to the clinic when she felt more comfortable. In other instances, providers mailed contraceptives or provided curbside pickup delivery for patients who expressed concerns or faced barriers with attending in-person appointments. One provider highlighted this commitment to patient-driven flexibility, saying *“I guess it really depends on what the patient is looking for, so I mean, we are always accessible if they want to be here, and if not, we will try to approach it the best way.”*

### 3.3. School-Based Staff Responded to Unique Challenges to Reach and Serve Youth

While many of the described adaptations occurred at both Title-X-funded and school-based clinics, school closures introduced unique challenges for school-based providers around reaching students, engaging in confidential conversations, and providing services on-site or in alternate locations. These challenges pushed family planning providers and staff in these settings to further adapt services and approaches to better meet the needs of their AYA patients.

In light of immediate and ongoing school closures, providers and staff in schools overwhelmingly spoke of challenges reaching students and informing them about the status of health care services and how to access them. One school-based provider said,

*It is a real challenge for anyone doing work with populations that have inadequate housing and are financially insecure. Phones being on and off and being able to contact patients, their numbers changing. I mean that is such a challenge for us, period.*

As a result, many providers and staff in school-based clinics, including health educators, described increased outreach efforts via phone calls, emails, or text messages. Some providers reported creating dedicated cell phone lines, such as a Google Voice line, to facilitate contact. In some cases, clinics also established social media platforms (e.g., Instagram or Facebook) or posted announcements on schools’ digital platforms to communicate with students and share information about their clinic hours, location(s), and services. In addition to this general outreach, providers also described conducting more targeted outreach to students who were on birth control or expressed interest in obtaining contraception. One school-based provider recounted how their clinic aimed to reach each student due for contraceptive injections or oral contraceptive refills, saying *“Every student that was due for Depo since we’ve been out has gotten a phone call or two or three from us to try to schedule them.”*

Providers in school settings contended with an additional layer of complexity given significant confidentiality and privacy concerns for AYA. During the early phases of the pandemic—with schools closed and lockdown in effect—students were likely at home with parents and other family members, which made communicating with clinic staff challenging, especially for those who did not have access to a personal cell phone or reliable internet service. One provider shared the difficult situation school-based staff were in, saying:

*It’s a really tough dance between respecting confidentiality and getting these kids services. I have students that have told me, ‘My parents absolutely cannot know about this.’ And they don’t have a cell phone of their own… I risk, even if I just call them, their parents asking them, ‘Why are they calling you?’, so I’ve really agonized a lot over what the right thing is.*

To address this, some staff discussed calling students in the late afternoons or evenings as this tended to be a time when AYA could talk confidentially. Other staff also shared that, if parents answered their outreach calls, staff would speak more generically about wellness services and encourage parents to share with the students to reach out to the clinic if they have questions or concerns.

Finally, school-based staff also faced barriers to providing services to students due to not having access to their clinic space or students being unable to secure transportation to be seen in person. In addition to providing telehealth and “curbside” services described above, school-based staff described using several unique strategies to continue services for school-based AYA, including providing mobile services, referring to or providing services at alternate locations, or sending prescriptions to nearby pharmacies. A small number of staff met students at offsite locations or students’ homes to provide needed services. One school-based provider described conducting contraceptive counseling via telehealth and then *“show[ing] up in our PPE and…dropping off their [prescription], whether it was asthma inhalers, the pills, the patch, or giving them their Depo shot in the car or in their house.”* In other cases, school-based staff partnered with outside organizations or local health departments to either refer students for care or provide services at those locations. For example, one provider reported arranging appointments for several students to receive their contraceptive injections at a neighboring occupational health site. Providers also described sending prescriptions to pharmacies that were close to students’ homes rather than asking students to collect their prescription at the school or clinic to mitigate transportation barriers.

### 3.4. COVID-19 Created Key Opportunities for Innovation

Interviews across both Title X and school-based sites revealed an overarching sense that, despite challenges, the rapid pace and unique context of the pandemic allowed for unprecedented creativity and innovation in family planning care. Providers and staff spoke of being pushed to consider new ways of providing services when standard practices were *“knocked out the window for the pandemic.”* Participants often expressed excitement or surprise at how well some adaptations worked or were received. For example, one school-based clinic director remarked about telehealth, *“Interestingly enough, that’ll probably be one of the things that lasts the longest. Because students like it. It’s accessible. It makes life easier…so I think that that’s going to be a wave of the future.”*

In many cases, participants described how some adaptations put in place because of the pandemic may have actually led to more streamlined and accessible services. Telehealth and digitized forms were often cited as changes that may persist due to important and unexpected benefits to staff and patients. For example, one Title X provider recounted realizing that obtaining medical histories over the phone was not only more efficient than doing so in person but also provided a level of anonymity that allowed patients to be more forthcoming. Another school-based staff member said of their pandemic-related switch to digital intake forms, *“Not only was it helpful for us being able to track things more, but I think it made it more accessible to our student body.”*

Ultimately, providers and staff expressed a feeling that COVID-19 broke down longstanding roadblocks or barriers that might not have been breached otherwise. One provider at a Title X clinic said of COVID-19, *“I think this has totally shaped, or actually changed, the way that a lot of medical providers have thought about providing medical care.”* In some cases, this was discussed as a mindset shift that could—or should—persist moving forward. For example, one Title X provider described how, for them, the surprise of how easily longstanding practices could be changed for the better will be a lasting effect of the pandemic:

*I think realizing that we have these barriers in place, whether it was, ‘Oh, you need to come in every 12 months for your birth control prescription renewal,’ or ‘No, we can’t. We’re not able to prescribe you that without a face-to-face visit.’ And those things were kind of unquestioned barriers that we all just sort of accepted. And then realizing how quickly they were able to bust through them. That’s what we really need to do.*

## 4. Discussion

This analysis expands previous research on family planning services during the COVID-19 pandemic by using in-depth interview data from providers and staff at Title X and school-based clinics to describe service adaptations in the early pandemic. These qualitative data build on existing descriptive information to provide key insights into provider experiences adapting family planning services, impressions on the usefulness and acceptability of these adaptations, and considerations for using these approaches moving forward, particularly in settings serving populations that face increased barriers to care. Our findings highlight the multiple, layered changes Title X providers and school-based clinic staff made to continue services, providers’ focus on ensuring flexible patient-centered care, the unique challenges of serving AYA in schools, and the potential for some COVID-19-related adaptations to change family planning service delivery for the better.

In exploring how providers adapted services, two approaches—telehealth and digitized administrative practices—emerged as potential long-term adaptations, both of which have important equity implications. Despite implementation challenges, telehealth services were largely embraced by providers and staff in our studies, with many noting their potential to expand access to care. This finding aligns with previous research with family planning clinicians [19,40,42]. In addition, many providers in our studies applauded use of online technology, including digitized forms that allowed patients to complete pre-visit screenings and intakes and thus streamline the amount of time patients spend in clinics. While promising, these practices raise several structural considerations for continued implementation. The ability to provide telehealth care for sexual and reproductive health is limited by federal and state laws and regulations, with wide variability across states [56]. In addition, telemedicine and digitized administrative procedures require client access to reliable internet and a location that ensures privacy, both of which can disproportionately be barriers for women from systematically marginalized groups and AYA, as well as individuals in rural communities [7,39,56]. It is critical that practitioners recognize that digital adaptations will not be accessible and beneficial for all patients. In many cases, providers will need to continue providing alternatives, such as in-person services or paper forms, to ensure equitable access to care. Research should also evaluate how these approaches benefit and are experienced by diverse patient populations, including AYA and those in areas where privacy or internet access are limited.

Interviews with school-based staff and providers highlighted additional considerations for practitioners serving AYA in school settings as the COVID-19 pandemic continues. Historically, an advantage of school-based health centers and programs is that they offer confidential care in the very place where AYA spend much of their time (versus requiring travel to an outside clinic). The early months of COVID-19 disrupted this model, causing school-based family planning providers to quickly assess how to maintain student contact and care. The concerns we heard around access to and confidentiality for family planning services for AYA have been noted by researchers and practitioners elsewhere [39,40,56]. Our data provide additional information on the ways in which staff mitigated these barriers, including increasing outreach efforts and providing services at alternate locations. It is possible that intensified outreach and service delivery efforts will persist through the ongoing pandemic, stretching already limited staff and resources. Ongoing partnerships and sharing of best practices among school-based staff will be necessary to sustain this work.

Finally, our findings point to a key success of family planning providers’ and staff response to COVID-19: their flexibility, resiliency, and innovation. This creativity and determination occurred against a backdrop of high rates of stress, burnout, and turnover among healthcare workers broadly [57,58,59]. Others have also noted this resilient response to the challenges of the pandemic [36,43]. However, our data further suggest a lasting eagerness among providers and staff to continue improving upon traditional clinical practices to ensure high-quality family planning care. As we enter subsequent phases of the pandemic, the family planning community may grapple with how to best encourage and allow for continued innovation. For example, many providers in our interviews reported adopting telehealth in response to COVID-19-initiated expanded coverage under Centers for Medicare and Medicaid [56]. Similarly, many described adjusting birth control spacing in response to FPNTC guidance on family planning care during COVID-19 [30]. Should these initiatives expire as the pandemic wanes, there may be a gap in supportive structures to scaffold innovation in clinical care.

While our data provide unique insight into the experiences of Title X and school-based providers and staff during a pivotal time period, some limitations must be noted. First and foremost, this analysis draws on combined subsets of data from two distinct but related projects that were not designed to be jointly analyzed. We opted to examine these data together due to several shared project features. For example, both projects engaged providers and staff working in settings that serve many women and adolescents who face barriers to accessing family planning care and used similar interview questions to explore service delivery at an inflection point of the COVID-19 pandemic. Analyzing these data together allowed us to examine similarities and differences between these two settings and gain a more holistic view of pandemic-era family planning care than if we had analyzed them separately. However, these samples and data were not uniform, and it is possible that key patterns were overweighted or overlooked. A second limitation is that interviews were only conducted with clinic providers and staff and do not incorporate client perspectives on service adaptations and clinical care. These perspectives are needed to provide important information on the extent to which service adaptations meet patient needs—context that is especially important for adaptations that are expected to continue, as discussed above. Moving forward, research should engage diverse patient populations, particularly those who typically experience greater barriers to access, to explore how they have experienced changes to practice and implications for equity in access to health care. Finally, interviews were conducted early in the pandemic and represent only one point in time in an ongoing event. Given the continuous pace of change noted by providers and staff, future studies should examine whether and how family planning delivery continues to evolve.

## 5. Conclusions

This paper explored provider and staff perspectives on adaptations made to family planning service delivery during the first year of the COVID-19 pandemic in Title-X-funded and school-based clinics—two settings that serve populations that experience barriers to accessing services. Interview data revealed that the pandemic demanded a layered, flexible approach from providers and staff to ensure continued high-quality services for women and AYA. Data also shed light on unique challenges faced by school-based staff serving AYA around reaching students and engaging them in confidential conversations when schools were closed. Ultimately, the findings suggest several lasting changes to family planning service delivery for populations hardest hit during the pandemic, including increased use of telehealth and digitized administrative procedures and a new openness to innovation among providers. We discuss considerations for family planning practitioners around ensuring equity in telehealth and digital health applications, serving school-based populations, and supporting provider resiliency and innovation. This analysis supports and provides important qualitative context to previous research on how providers continued providing critical family planning services during the early pandemic. Future studies are needed to evaluate promising practices, such as telehealth and digitized administration, and explore how these are perceived by diverse patient populations that face barriers to accessing care, particularly AYA and those in areas where privacy or internet access are limited.

## Figures and Tables

**Table 2:** Service delivery adaptations described by Title X and school-based staff.

Adaptation	Description	Provider and Staff Impressions	Provider/Staff Quote
Telehealth	Using phone or video calls to screen clients for COVID-19, triage whether a client needed an in-person appointment, conduct contraceptive counseling, start a client on a contraceptive method, and hold follow-up conversations.	Staff described multiple benefits to seeing patients by phone or video, including increased access to services and convenience for patients. Some providers mentioned issues with clients not having adequate internet bandwidth, devices, or data plans to support video calls; others noted that the practice does not replace face-to-face interactions. However, most providers spoke positively of telehealth and felt that it will be an important complement to in-person care moving forward. Some providers and staff were unsure whether Medicaid coverage for telehealth, which many states expanded during the pandemic, would continue [48].	*We do have telehealth. We’ve had that through most of the pandemic. It took a little bit to get it going, but now we have a pretty robust system, and we have a hotline that anyone under 19 can call. Parents, kids, school staff can reach us, and either have a full telehealth visit or just ask questions, or ask for a med refill, anything they need. And we have a behavioral health person staffing that every day too. So a pretty good system there.* (School-Based Provider)
Prioritizing urgent services	Prioritizing certain services and procedures, such as colposcopies and LARC insertions, for in-person care while delaying or remotely delivering routine care, such as annual exams.	Prioritizing services was seen as a temporary measure, used more often in the early months of the pandemic in response to staffing shortages or social distancing requirements. Even with key services being prioritized, the overall reduced numbers of in-person appointments available often led to extended wait times for in-person LARC appointments. In these cases, providers typically offered patients bridge methods of birth control, such as the pill, patch, or ring. Multiple staff described getting “back to routine” in later months.	*[Annual exams] were postponed for patient safety. People that had abnormal paps, etcetera. those people came in but if it was a very healthy individual, and they were coming in just to get their annual and their birth control re-filled, we just gave them their birth control refill.* (Title X Administrator and Provider)
“Curbside” services	Delivering services and/or prescriptions outside or in a drive-by fashion; most commonly used to administer contraceptive injections, distribute other contraceptives, such as pills, and conduct COVID-19 screenings.	Staff felt that patients appreciated the ease and convenience of curbside services; a few noted a decrease in “no-shows” for curbside appointments compared to standard appointments. Some clinics did not offer this service due to concerns over cleanliness, safety, or the potential for HIPAA violations. Most felt that the practice would not continue post-pandemic.	*Something that we did develop with COVID was doing our follow-ups for family [planning visits] and Depo—we’re following up through telemedicine, and then we’re doing a curbside. They’re just coming in for that last piece to sign the updated consent and do the Depo.* (School-Based Provider)
Flexible approaches to birth control refills and spacing	Extending the interval required for in-office visits for birth control prescription refills; prolonging the time required between contraceptive injections; providing subcutaneous contraceptive injection refills for self-administration.	Staff often made these adaptations following guidance from the Family Planning National Training Center’s (FPNTC) guidance document on spacing for oral contraceptives and contraceptive injections [24]. Many staff felt that the increased flexibility around contraceptive refills and injections worked well for both providers and patients and should be integrated into practice moving forward.	*The biggest problem was our annual visits. You’re coming for your annual visit so you can get a refill of, like, birth control pills. So those people, what we did is because we didn’t know what was going on, how long it was going to take, our standard [was to give] them an extra two months of birth control, have them reschedule for two months out, make sure that they’re not having any problems and they don’t truly need to see the nurse practitioner or the nurse at that time.* (Title X Provider)
Streamlined administrative processes	Using phone or digital technology (video calls, online portals, and digital forms) to reduce the amount of time clients spent on non-medical activities inside clinics; includes streamlining sign-ins, medical history forms, and pre-visit screenings and intake forms.	Multiple providers and staff noted that moving to digital or phone-based intakes, sign-ins, and forms had improved efficiency and clinic flow, enabling more time spent delivering care. Many staff expressed a desire for these streamlined processes to continue post-pandemic.	*[Collecting intake information over the phone] makes things very efficient in the clinic. So we’ve done the intake over the phone, and we can get them in and do their vitals, and they get right straight in front of the practitioner…If we can do intakes over the phone when appropriate, we may continue that*. (Title X Administrator)

## Data Availability

Data for this analysis are not publicly available due to the need to protect the privacy of the research participants. Requests for deidentified data to support study findings should be directed to the corresponding author.
